# Psychological Impacts of COVID-19 During the First Nationwide Lockdown in Vietnam: Web-Based, Cross-Sectional Survey Study

**DOI:** 10.2196/24776

**Published:** 2020-12-15

**Authors:** Khanh Ngoc Cong Duong, Tien Nguyen Le Bao, Phuong Thi Lan Nguyen, Thanh Vo Van, Toi Phung Lam, Anh Pham Gia, Luerat Anuratpanich, Bay Vo Van

**Affiliations:** 1 Mahidol University Health Technology Assessment (MUHTA) Graduate Program Mahidol University Bangkok Thailand; 2 Institute of Orthopaedics and Trauma Surgery Viet Duc Hospital Hanoi Vietnam; 3 Social Economics and Administrative Pharmacy Program, Department of Pharmacy Faculty of Pharmacy Mahidol University Bangkok Thailand; 4 Department of Surgery Hanoi Medical University Hanoi Vietnam; 5 Health Strategy and Policy Institute Ministry of Health Hanoi Vietnam; 6 Oncology Department Viet Duc Hospital Hanoi Vietnam; 7 Department of Pharmacy Thong Nhat Hospital Ho Chi Minh City Vietnam

**Keywords:** COVID-19, mental health, psychological distress, depression, anxiety, Vietnam, psychology, distress, lockdown, survey

## Abstract

**Background:**

The first nationwide lockdown due to the COVID-19 pandemic was implemented in Vietnam from April 1 to 15, 2020. Nevertheless, there has been limited information on the impact of COVID-19 on the psychological health of the public.

**Objective:**

This study aimed to estimate the prevalence of psychological issues and identify the factors associated with the psychological impact of COVID-19 during the first nationwide lockdown among the general population in Vietnam.

**Methods:**

We employed a cross-sectional study design with convenience sampling. A self-administered, online survey was used to collect data and assess psychological distress, depression, anxiety, and stress of participants from April 10 to 15, 2020. The Impact of Event Scale-Revised (IES-R) and the Depression, Anxiety, and Stress Scale-21 (DASS-21) were utilized to assess psychological distress, depression, anxiety, and stress of participants during social distancing due to COVID-19. Associations across factors were explored using regression analysis.

**Results:**

A total of 1385 respondents completed the survey. Of this, 35.9% (n=497) experienced psychological distress, as well as depression (n=325, 23.5%), anxiety (n=195, 14.1%), and stress (n=309, 22.3%). Respondents who evaluated their physical health as average had a higher IES-R score (beta coefficient [B]=9.16, 95% CI 6.43 to 11.89), as well as higher depression (B=5.85, 95% CI 4.49 to 7.21), anxiety (B=3.64, 95% CI 2.64 to 4.63), and stress (B=5.19, 95% CI 3.83 to 6.56) scores for DASS-21 than those who rated their health as good or very good. Those who self-reported their health as bad or very bad experienced more severe depression (B=9.57, 95% CI 4.54 to 14.59), anxiety (B=7.24, 95% CI 3.55 to 10.9), and stress (B=10.60, 95% CI 5.56 to 15.65). Unemployment was more likely to be associated with depression (B=3.34, 95% CI 1.68 to 5.01) and stress (B=2.34, 95% CI 0.84 to 3.85). Regarding worries about COVID-19, more than half (n=755, 54.5%) expressed concern for their children aged <18 years, which increased their IES-R score (B=7.81, 95% CI 4.98 to 10.64) and DASS-21 stress score (B=1.75, 95% CI 0.27 to 3.24). The majority of respondents (n=1335, 96.4%) were confident about their doctor’s expertise in terms of COVID-19 diagnosis and treatment, which was positively associated with less distress caused by the outbreak (B=–7.84, 95% CI –14.58 to –1.11).

**Conclusions:**

The findings highlight the effect of COVID-19 on mental health during the nationwide lockdown among the general population in Vietnam. The study provides useful evidence for policy decision makers to develop and implement interventions to mitigate these impacts.

## Introduction

COVID-19 was first reported in December 2019 in China. It was then declared as a pandemic on March 11, 2020 [1]. Globally, there were 58,087 confirmed cases and up to 3670 fatalities, as of March 31, 2020 [2].

Vietnam detected its first two COVID-19 cases on January 22, 2020 [3]. Since then, the country has faced an increased likelihood of infection spread due to its proximity to China and its low-resource health care system. Responding to this urgency, the Vietnamese government declared COVID-19 as an epidemic on February 1, 2020. It implemented proactive actions to confront the spread of the disease [4]. The number of cases, however, increased approximately by 15-folds in 2 weeks from 15 cases on March 15, 2020, to 206 cases on March 31, 2020 [5]. Notably, the epicenter was the largest public hospital in Hanoi, Vietnam [6]. Accordingly, the government imposed the first nationwide lockdown from April 1 to 15, 2020 [7]. It was a partial lockdown that required people to stay at home (with exceptions made for the purchase of critical supplies, health emergencies, and essential workers and businesses) along with personal protective measures such as wearing face masks in public places and hand washing [7]. As the government has clearly and frequently communicated with the public about the dangers of the illness as well as preventive measures through text messages and social media, citizens are aware of the complications of the pandemic, and thus voluntarily abide regulations [8].

Several social distancing measures, such as home quarantine, school closures, and nonessential business closures, have caused negative psychological effects on populations, including depression, anxiety, and posttraumatic distress, which were also found in previous epidemics [9]. Nguyen et al [10] surveyed the early stage of the pandemic from February 14 to March 13, 2020, and found that patients with suspected COVID-19 symptoms were 2.88 times (95% CI 2.18 to 3.80) more likely to experience depression and significantly lower health-related quality of life (HRQOL) (*P*<.001) as compared with those without suspected COVID-19 symptoms. The study also reported that the prevalence of depression was 7.4% [10]. With dramatically increasing COVID-19 cases and a compulsory nationwide lockdown, it is anticipated that the prevalence of mental health issues in the general population in Vietnam would surge considerably. We therefore conducted this study to investigate the psychological impacts of COVID-19 among the general population in Vietnam during the first national lockdown and to identify the associated factors of these impacts.

## Methods

### Participants and Procedure

A cross-sectional study design was employed using a respondent-driven sampling method by distributing a self-administered survey through various social media platforms (eg, Facebook, Zalo, etc) from April 10 to 15, 2020, in Vietnam. All Vietnamese residents who were aged ≥18 years and provided informed consent were eligible to participate in the online survey.

The starting point for data collection was 1 week following the implementation of the national lockdown (April 1, 2020) to ensure that participants had been exposed to a sufficient amount of time to social distancing in order to properly measure psychological impacts over the past week [11,12]. A written informed consent was received online before the respondents answered the questionnaire. They clicked the link on the survey platform and voluntarily responded to the survey. Anonymity and confidentiality were ensured throughout the conduct of the survey.

### Questionnaire

We employed a self-administered questionnaire, which consisted of three parts and could be completed in 10 minutes. The first part covered the general sociodemographic characteristics of the participants, including age, gender, area of residence, education, marital status, income, occupation, history of chronic disease, household size, current physical health status, and other basic information. The second part was composed of questions related to concerns about the COVID-19 pandemic, which were adapted from a previous study [13]. The third part of the survey included the two psychological and mental health measurement scales described below.

#### Impact of Event Scale-Revised

The Impact of Event Scale-Revised (IES-R) has 22 items that measure psychological distress after exposure to a crisis. The total IES-R score was categorized as normal (0-23), mild (24-32), moderate (33-36), and severe (≥37) [14]. The IES-R questionnaire has been used in the Vietnamese population in previous studies [14-16]. In this study, a score of ≥24 signified a positive cutoff point to estimate distress due to the COVID-19 lockdown [11,17].

#### Depression, Anxiety, and Stress Scale-21

Impacts on mental health, including depression, anxiety, and stress, were assessed using the Depression, Anxiety, and Stress Scale-21 (DASS-21). The questionnaire consisted of 21 items that covered three domains: depression, anxiety, and stress. The score calculation for each domain was adapted from a previous study [18]. The total score range for each domain was 0 to 42. The level of each domain was interpreted as normal (0-9), mild (10-12), moderate (13-20), severe (21-27), and extremely severe (28-42) for the depression subscale; normal (0-6), mild (7-9), moderate (10-14), severe (15-19), and extremely severe (20-42) for the anxiety subscale; and normal (0-10), mild (11-18), moderate (19-26), severe (27-34), and extremely severe (35-42) for the stress subscale. Cutoff scores of 9, 6, and 10 for the depression, anxiety, and stress subscales, respectively, were used to detect depression, anxiety, and stress. The DASS-21 questionnaire has been assessed for reliability and validity in the Vietnamese context previously [19,20].

The data collection tool was piloted in 10 individuals to further develop the questionnaire, and a few questions were adjusted in terms of language and idea expression.

### Data Analysis

Descriptive statistics were used to present sociodemographic characteristics. Continuous variables were presented as mean and standard deviation if the data were normally distributed, or median and interquartile range for nonnormal distribution. To identify variables that could be associated with psychological distress, depression, anxiety, and stress, univariate analyses were performed. Subsequently, all variables with a *P*<.20 in the analyses were entered into the multivariate regression model [21]. The association between factors and IES-R score, and DASS-21 depression, anxiety, and stress subscale scores, were reported with beta coefficients (B) and 95% CIs. *P*<.05 was set as the level of statistical significance. All of the statistical analyses were performed using R software, version 3.6.3 (The R Project for Statistical Computing) [22].

This online survey was reported in accordance with CHERRIES (Checklist for Reporting Results of Internet E-Surveys) [23] (Multimedia Appendix 1).

### Ethics Statement

The study protocol was approved by the Council of Medical Ethics at Thong Nhat Hospital in Ho Chi Minh City, Vietnam (number 10/BB-BVTN).

## Results

A total of 1412 participants filled out the questionnaire. Of these, 1385 (98.1%) were valid for analysis. Reasons for exclusion were invalid age information (n=8), age <18 years (n=10), and respondents living outside Vietnam (n=9).

The majority of the respondents were male (n=880, 63.5%). The median age was 28 years (range 18-70 years), with 82.2% (n=1139) aged 18-39 years. In terms of area of residence, 73.0% (n=1011) lived in urban areas and 30.3% (n=419) lived in provinces or cities with COVID-19 cases. The majority obtained an undergraduate or higher degree (n=1174, 84.8%). As for employment, most were employed during the lockdown (n=895, 64.6%); a few worked from home (n=446, 32.2%) and some were unemployed (n=113, 8.2%). Most respondents (n=1243, 89.7%) had at least one chronic disease. Additional sociodemographic characteristics of participants are presented in Multimedia Appendix 2.

The total IES-R score ranged from 0 to 88 with a median of 17 (IQR 20). The DASS-21 scores ranged from 0 to 42 (median 2, IQR 8) for depression, 0 to 38 (median 0, IQR 4) for anxiety, and 0 to 42 (median 0, IQR 10) for stress. Using the cutoff points, we found that 35.9% (n=497) had psychological distress. In addition, respondents experienced symptoms of depression (n=325, 23.5%), anxiety (n=195, 14.1%), and stress (n=309, 22.3%), respectively. These results are displayed in Multimedia Appendix 3.

In multivariate regression analysis, associations were found between IES-R scores and variables marital status and health status. Respondents who were married, divorced, or widowed had higher IES-R scores compared with those who were single (B=2.60, 95% CI 0.51 to 4.69 vs B=5.23, 95% CI 0.50 to 9.95). Individuals who self-evaluated their physical health as average had higher psychological distress than those who reported good or very good health (B=9.16, 95% CI 6.43 to 11.89) (Table 1).

**Table 1:** Multivariate linear regression results for the Impact of Event Scale-Revised questionnaire scores and sociodemographic covariates.

Covariates		Coefficient (95% CI)	*P* value
**Age group (years) (reference: 18-39)**			
	40-59	1.23 (–1.13 to 3.58)	.31
	≥60	–8.36 (–17.72 to 10)	.08
**Marital status (reference: single)**			
	Married	2.60 (0.51 to 4.69)	.01
	Divorced/widowed	5.23 (0.50 to 9.95)	.03
**Education level (reference: elementary/secondary)**			
	High school	3.20 (–4.18 to 10.58)	.40
	University/college	5.60 (–1.51 to 12.71)	.12
	Postgraduate	4.40 (–2.93 to 11.74)	.24
**Occupation (reference: employed)**			
	Work from home	0.95 (–1.24 to 3.14)	.40
	Student	0.98 (–2.15 to 4.11)	.54
	Unemployed	3.32 (–0.01 to 6.65)	.051
	Other	1.37 (–3.17 to 5.91)	.55
**Household size (reference: 1 member)**			
	2	3.86 (–0.05 to 7.77)	.05
	3-5	1.65 (–1.65 to 4.95)	.33
	≥6	2.24 (–1.61 to 6.10)	.25
**Children <18 years old in the family (reference: no)**			
	Yes	1.02 (–0.80 to 2.83)	.27
**Chronic disease (reference: no)**			
	Yes	1.38 (–1.23 to 40)	.30
**Average income per month (million VND^a^) (reference: no income)**			
	<1	1.92 (–3.34 to 7.18)	.47
	1-5	–0.17 (–3.27 to 2.94)	.91
	5-10	1.53 (–1.46 to 4.53)	.32
	10-20	–1.12 (–4.34 to 2.10)	.49
	>20	–0.22 (–3.75 to 3.32)	.90
**Average length of stay (hours) at home during the lockdown (reference: 0-10)**			
	11-20	–0.94 (–3.53 to 1.65)	.48
	21-24	–2.74 (–5.60 to 0.12)	.06
**Current health status (reference: good/very good)**			
	Average	9.16 (6.43 to 11.89)	<.001
	Bad/very bad	8.72 (–1.38 to 18.81)	.09

^a^VND: Vietnam Dong.

Age, marital status, occupation, chronic disease, physical health conditions, and residents in areas with infected cases were found to be associated with depression in the multiple regression model. People aged ≥60 years were less depressed than people aged 18-39 years (B=–5.86, 95% CI –10.52 to –1.20). Married respondents had smaller depression scores than their single counterparts (B=–1.02, 95% CI –2.00 to –0.03). Those who were unemployed (B=3.34, 95% CI 1.68 to 5.01), students (B=1.76, 95% CI 0.19 to 3.32), and those who worked from home (B=1.33, 95% CI 0.24 to 2.43) had a higher risk of depression compared to employed participants. Having chronic disease was associated with higher depression (B=1.32, 95% CI 0.02 to 2.63). People with an average, bad, or very bad physical health self-assessment exhibited higher depression scores than those who reported good or very good health (B=5.85, 95% CI 4.49 to 7.21 vs B=9.57, 95% CI 4.54 to 14.59). People residing in provinces or cities with COVID-19 cases had an elevated risk for depression (B=1.69, 95% CI 0.83 to 2.55) compared with those who lived elsewhere (Table 2).

**Table 2:** Multivariate linear regression results for the Depression, Anxiety, and Stress Scale-21 (depression subscale) with sociodemographic covariates.

Covariates		Coefficient (95% CI)	*P* value
**Age group (years) (reference: 18-39)**			
	40-59	–0.12 (–1.29 to 1.05)	.84
	≥60	–5.86 (–10.52 to –1.20)	.01
**Marital status (reference: single)**			
	Married	–1.02 (–2.00 to –0.03)	.04
	Divorced/widowed	0.41 (–1.90 to 2.73)	.73
**Education level (reference: elementary/secondary)**			
	High school	–2.74 (–6.41 to 0.94)	.14
	University/college	–2.65 (–6.19 to 0.89)	.14
	Postgraduate	–2.39 (–6.05 to 1.26)	.20
**Occupation (reference: employed)**			
	Work from home	1.33 (0.24 to 2.43)	.02
	Student	1.76 (0.19 to 3.32)	.03
	Unemployed	3.34 (1.68 to 5.01)	<.001
	Other	2.11 (–0.15 to 4.37)	.07
**Chronic disease (reference: no)**			
	Yes	1.32 (0.02 to 2.63)	.047
**Current situation (reference: social distancing)**			
	Quarantine/isolation	0.62 (–0.91 to 2.15)	.43
**Average income per month (million VND ^a^) (reference: no income)**			
	<1	0.16 (–2.46 to 2.78)	.90
	1-5	–0.54 (–2.09 to 1.00)	.49
	5-10	–0.15 (–1.64 to 1.35)	.84
	10-20	–0.79 (–2.40 to 0.82)	.34
	>20	–0.08 (–1.85 to 1.70)	.93
**Average length of stay (hours) at home during the lockdown (reference: 0-10)**			
	11-20	–0.65 (–1.94 to 0.64)	.33
	21-24	–0.38 (–1.8 to 1.05)	.60
**Current health status (reference: good/very good)**			
	Average	5.85 (4.49 to 7.21)	<.001
	Bad/very bad	9.57 (4.54 to 14.59)	<.001
**Infected cases in province/city (reference: no)**			
	Yes	1.69 (0.83 to 2.55)	<.001

^a^VND: Vietnam Dong.

The association between respondents’ isolation situation and current health self-assessment with symptoms of anxiety remained associated in the multivariate analysis. Isolated participants (at quarantine centers or at home) had a higher likelihood of experiencing anxiety (B=1.22, 95% CI 0.11 to 2.33). The respondents who rated their health as average (B=3.64, 95% CI 2.64 to 4.63) and bad or very bad (B=7.24, 95% CI 3.55 to 10.90) were more likely to exhibit anxiety than those who reported good or very good health (Multimedia Appendix 4).

Occupation, physical health condition, and COVID-19 cases in place of residence were factors associated with symptoms of stress among respondents. Being unemployed or a student was associated with more stress than employed individuals (B=2.34, 95% CI 0.84 to 3.85 vs B=1.17, 95% CI 0.10 to 2.23). Participants with average (B=5.19, 95% CI 3.83 to 6.56) and very bad or bad (B=10.60, 95% CI 5.56 to 15.65) health evaluations demonstrated a higher risk for stress than those with a good or very good self-reported health status. A higher stress score was recorded among respondents who were living in areas with COVID-19 cases (B=0.92, 95% CI 0.08 to 1.76) (Multimedia Appendix 5).

The results for univariate linear regression for the IES-R and DASS-21 scales and sociodemographic factors are presented in Multimedia Appendix 6.

We found that 96.4% (n=1335) of Vietnamese residents were confident of their doctor’s expertise on COVID-19 diagnosis and treatment. Most believed that they were at a low likelihood of contracting COVID-19 (n=841, 63.3%) and perceived that they could survive if they were infected (n=1189, 85.9%). More than half expressed concerns for their children who were under the age of 18 years (n=755, 54.5%) as well as for other family members (n=892, 64.5%). In the multivariate regression analysis, the likelihood of surviving if infected remained statistically associated with the DASS-21 depression and anxiety subscales. Having concerns about children aged <18 years were associated with IES-R score and DASS-21 stress. Respondents who held a particular belief about their likelihood of surviving if infected (ie, somewhat likely, not very likely, and not at all likely) experienced more depression than those who did not know their likelihood (B=2.07, 95% CI 0.59 to 3.55, B=4.10, 95% CI 1.47 to 6.72 vs B=8.67, 95% CI 0.14 to 17.19). In addition, those who believed that they were somewhat likely to survive if infected with COVID-19 were more anxious than those who did not know their likelihood (B=1.21, 95% CI 0.15 to 2.27). Those who were worried about their children aged <18 years had increased IES-R and DASS-21 stress scores than those without children aged <18 years (B=7.81, 95% CI 4.98 to 10.64 vs B=1.75, 95% CI 0.27 to 3.24). Participants who were somewhat worried about children aged <18 years also experienced greater psychological distress (B=3.19, 95% CI 0.61, 5.78). The results for the analysis of the association between concerns related to the COVID-19 pandemic and psychological distress, depression, anxiety, and stress are shown in Multimedia Appendices 7 and 8.

## Discussion

### Principal Findings

Our study investigated in detail the psychological and mental health impacts of the COVID-19 pandemic on the general population in Vietnam during the first nationwide lockdown. The findings revealed that more than one-third of respondents experienced psychological distress (35.9%), nearly one-quarter exhibited depression and stress (23.5% and 22.3%, respectively), and 14.1% experienced anxiety. The prevalence of the depressed population in the present study was higher than that reported by Nguyen et al [10]; their study investigated outpatients in hospitals and health centers in Vietnam from February 14 to March 13, 2020, and found that 7.4% of the sample exhibited symptoms of depression. There may be some explanations for this discrepancy. First, in February, Vietnam had not implemented compulsory social distancing at the national level while strictly large-scale social distancing substantially increased anxiety, depression, loneliness, and substance use [24]. Second, our questionnaire was distributed via social media platforms, which might involve people who were more concerned about the pandemic and thus there might be a high probability of exposure to misinformation and fake news that could elevate depression rates [25]. Moreover, different measurement scales could be attributed to different prevalence.

Our research findings also suggested factors associated with psychological and mental health impact, which may identify vulnerable populations, such as unemployed individuals, those with a bad or very bad self-reported physical health status, or those living in areas with COVID-19 cases.

Our results confirmed previous research, which found that employment could be a protective factor for mental health during the pandemic [26]. Additionally, participants working from home were more prone to depression than those who returned to their workplaces. This could be due to reduced social interactions and more distractions from pandemic-related news among people working from home, which might increase their depression. Unemployed people were deemed to have the worst psychological and mental health status among occupations in our study, experiencing distress, depression, and anxiety. Indeed, the Vietnamese government has implemented some interventions for this vulnerable population, such as an interim relief package (63 trillion VND) for specific people during the pandemic (eg, furloughed workers, unpaid leave workers, freelancers, the poor, or near-poor individuals, etc) [4]. These short-term interventions could partly mitigate socioeconomic burden. However, to ensure socioeconomic stability during future pandemics, long-term interventions, such as strengthening health care systems and having social protection schemes, should be taken into consideration [4].

Previous studies have shown that poor health status was a negative factor for mental health during the pandemic [10,13,27], which was also seen in our study. Notably speaking, residents in provinces or cities where there were COVID-19 cases were more sensitive to depression and stress. This increase in depression could be influenced by the fear of contracting an infection by living in higher-risk areas. Shi et al [26] showed that people living in higher-risk areas (Hubei) significantly experienced depression, anxiety, insomnia, and acute stress than those living in other areas. This may suggest that public health interventions should be focused on these vulnerable areas.

Nguyen et al [10] revealed that married individuals expressed a lower HRQOL during the COVID-19 pandemic, implying that married participants experienced psychological distress during the COVID-19 lockdown [10]; this was also seen in our study. However, married individuals tended to have lower depression than single individuals during this period. This could be explained by the strong association between loneliness and depression during the lockdown, where married people were less lonely than single people [28].

In terms of concerns related to COVID-19, the majority of participants (n=1189, 85.8%) perceived a high likelihood of surviving if they were infected with COVID-19. This was consistent with a prior study [13]. This can be inferred from their belief in their doctors’ capacity to treat COVID-19; in fact, more than 95% (n=1335) of Vietnamese citizens were somewhat or very confident about their health care professionals. This can lead to a decrease in the public’s distress level during the pandemic. Another positive finding observed was that those who perceived a reduced likelihood of contracting COVID-19 exhibited less distress during the lockdown. These findings point to the benefits of using contact tracing apps, such as Bluezone [29]. Not only does this intervention effectively support the epidemiological investigation, but it also reduces anxiety among those who were more likely to get exposed to COVID-19 (eg, taxi drivers).

With regards to concerns about family members, people with children younger than 18 years worried more and experience heightened distress, anxiety, and stress. This reflects Vietnamese culture as citizens often live in a three-generation household. Therefore, community interventions that aim to enhance health awareness in order to reduce risk and promote resilience among vulnerable populations are needed. These actions can also help strengthen our long-term response to the pandemic and prepare more effectively for future public health emergencies [30,31]. It suggests that providing reliable and accurate health information through health education or consulting programs, targeted at protecting children and the elderly, could help decrease the mental health impact of the lockdown on family members [32,33].

### Strengths and Limitations

This study had some strengths. First, it provided comprehensive information on the prevalence of psychological and mental health impacts of COVID-19 during the first nationwide lockdown. It used IES-R to measure the public crisis impact of the lockdown, and DASS-21 to capture the population’s depression, anxiety, and stress levels. Both scales complemented each other to provide a comprehensive picture of the psychological and mental health impact of the first nationwide lockdown in Vietnam. Second, we investigated the public’s concerns on COVID-19, which played a useful role in determining which public health interventions might be implemented to mitigate the impact. Third, areas with COVID-19 infections were also pointed out in our findings as vulnerable areas, where citizens may be more likely to experience mental health issues. This suggests that more action plans should be considered in these areas, which could control the disease effectively and reduce psychological impacts.

Several limitations existed in the study as well. First, the online survey with convenience sampling might limit the representativeness of the study. Second, measurement tools were self-assessed by participants, which might possess social bias. Third, the study design was cross-sectional, which reflects the population at the study time point and limits a causal relationship. However, in the context of nationwide social distancing, which restricted face-to-face interviews, and the need to carry out data collection in a short time period, these limitations were inevitable. Regardless, further studies are needed to assess possible long-term psychological and mental health impacts of COVID-19 on the population. In addition, as social distancing during the pandemic has been implemented in many countries and the use of web-based tools to measure psychological impacts has been increasing [34,35], more studies in this field are needed to evaluate these tools.

## Appendix

Multimedia Appendix 1 Checklist for Reporting Results of Internet E-Surveys (CHERRIES).

Multimedia Appendix 2 Sociodemographic characteristics of survey respondents.

Multimedia Appendix 3 Prevalence and score of the psychological and mental health impacts of COVID-19 on the general population in Vietnam during the first lockdown.

Multimedia Appendix 4 Multivariate linear regression results for the Depression, Anxiety, and Stress Scale-21 (anxiety subscale) with sociodemographic covariates.

Multimedia Appendix 5 Multivariate linear regression results for the Depression, Anxiety, and Stress Scale-21 (stress subscale) with sociodemographic covariates.

Multimedia Appendix 6 Univariate linear regression results for the Impact of Event Scale-Revised and the Depression, Anxiety, and Stress Scale-21 with sociodemographic covariates.

Multimedia Appendix 7 Univariate linear regression results for the Impact of Event Scale-Revised and the Depression, Anxiety, and Stress Scale-21 with concern-related covariates.

Multimedia Appendix 8 Multivariate linear regression results for the Impact of Event Scale-Revised and the Depression, Anxiety, and Stress Scale-21 with concern-related covariates.

## Abbreviations

CHERRIES: Checklist for Reporting Results of Internet E-Surveys
DASS-21: Depression, Anxiety, and Stress Scale-21
HRQOL: health-related quality of life
IES-R: Impact of Event Scale-Revised
