# Correction: Psychological Impacts of COVID-19 During the First Nationwide Lockdown in Vietnam: Web-Based, Cross-Sectional Survey Study

**DOI:** 10.2196/28357

**Published:** 2021-03-05

**Authors:** Khanh Ngoc Cong Duong, Tien Nguyen Le Bao, Phuong Thi Lan Nguyen, Thanh Vo Van, Toi Phung Lam, Anh Pham Gia, Luerat Anuratpanich, Bay Vo Van

**Affiliations:** 1 Mahidol University Health Technology Assessment (MUHTA) Graduate Program Mahidol University Bangkok Thailand; 2 Institute of Orthopaedics and Trauma Surgery Viet Duc Hospital Hanoi Vietnam; 3 Social Economics and Administrative Pharmacy Program, Department of Pharmacy Faculty of Pharmacy Mahidol University Bangkok Thailand; 4 Department of Surgery Hanoi Medical University Hanoi Vietnam; 5 Health Strategy and Policy Institute Ministry of Health Hanoi Vietnam; 6 Oncology Department Viet Duc Hospital Hanoi Vietnam; 7 Department of Pharmacy Thong Nhat Hospital Ho Chi Minh City Vietnam

In “Psychological Impacts of COVID-19 During the First Nationwide Lockdown in Vietnam: Web-Based, Cross-Sectional Survey Study” (JMIR Form Res 2020;4(12):e24776) three errors were noted.

In the originally published paper, the “greater than or equal to” symbol (≥) was missing in three places in tables due to an XML conversion error. The following corrections have been made:

In Table 1, under “Age group (years) (reference: 18-39)," “60” has been corrected to “≥60”.

In Table 1, under “Household size (reference: 1 member)," “6” has been corrected to “≥6”.

In Table 2, under “Age group (years) (reference: 18-39)," “60” has been corrected to “≥60”.

The correction will appear in the online version of the paper on the JMIR Publications website on March 5, 2021, together with the publication of this correction notice. Because this was made after submission to PubMed, PubMed Central, and other full-text repositories, the corrected article has also been resubmitted to those repositories.

