# Supplementary material for: Psychological Impacts of COVID-19 During the First Nationwide Lockdown in Vietnam: Web-Based, Cross-Sectional Survey Study
Source: JMIR Form Res. 2020 Dec 15;4(12):e24776. doi: 10.2196/24776 (PMC7935248; doi:10.2196/24776)
Supplement: Multimedia Appendix 1 [file formative_v4i12e24776_app1.doc]

**Multimedia Appendix 1: Checklist for Reporting Results of Internet E-Surveys (CHERRIES)**

| ***Checklist Item*** | ***Explanation*** | ***In the Manuscript*** | ***Details of Explanation*** |
| --- | --- | --- | --- |
| Describe survey design | Describe target population, sample frame. Is the sample a convenience sample? (In “open” surveys this is most likely.) | Methods: Participants and Procedure | “All Vietnamese residents who were aged 18 years and above, had signed informed consent and had voluntarily participated in the online survey during the study period were eligible for the survey.” |
| IRB approval | Mention whether the study has been approved by an IRB. | Ethics statement | “The study protocol was approved by the Council of Medical Ethics at Thong Nhat Hospital at Ho Chi Minh City, Vietnam (Number 10/BB-BVTN)” |
| Informed consent | Describe the informed consent process. Where were the participants told the length of time of the survey, which data were stored and where and for how long, who the investigator was, and the purpose of the study? | Method: Participants and Procedure | “All Vietnamese residents who were aged 18 years and above, had signed informed consent and had voluntarily participated in the online survey during the study period were eligible for the survey.”  “A written informed consent was received online before the respondents answered the questionnaire. They clicked the link on the platforms and voluntarily responded to the survey. Anonymity and confidentiality were ensured throughout the conduct of the survey.” |
| Data protection | If any personal information was collected or stored, describe what mechanisms were used to protect unauthorized access. | Method: Participants and Procedure | No personal identifying information was collected  The data of survey were stored on a secure server.  “Anonymity and confidentiality were ensured throughout the conduct of the survey.” |
| Development and testing | State how the survey was developed, including whether the usability and technical functionality of the electronic questionnaire had been tested before fielding the questionnaire. | Method: Measurement Instrument | “The instrument is a self-administered questionnaire which consisted of three parts and could be completed in 10 minutes….”  “The data collection tool was piloted in ten individuals to further develop the questionnaire. Consequently, few questions were adjusted in terms of language and idea expression.” |
| Open survey versus closed survey | An “open survey” is a survey open for each visitor of a site, while a closed survey is only open to a sample which the investigator knows (password-protected survey). | Method: Participants and Procedure | This study is an open survey |
| Contact mode | Indicate whether or not the initial contact with the potential participants was made on the Internet. (Investigators may also send out questionnaires by mail and allow for Web-based data entry.) | Method: Participants and Procedure | Banner ads for the study were posted through various social media platforms (e.g., Facebook, Zalo, etc.). No contact was made with participants outside of the internet-based survey  “A cross-sectional study design was employed using a respondent driven sampling method by distributing a self-administered survey through various social media platforms (e.g., Facebook, Zalo, etc.) from April 10 to 15, 2020 in Vietnam.” |
| Advertising the survey | How/where was the survey announced or advertised? Some examples are offline media (newspapers), or online (mailing lists – If yes, which ones?) or banner ads (Where were these banner ads posted and what did they look like?). It is important to know the wording of the announcement as it will heavily influence who chooses to participate. Ideally the survey announcement should be published as an appendix. | Method: Participants and Procedure | Banner ads for the study were posted through various social media platforms (e.g., Facebook, Zalo, etc.).  “A cross-sectional study design was employed using a respondent driven sampling method by distributing a self-administered survey through various social media platforms (e.g., Facebook, Zalo, etc.) from April 10 to 15, 2020 in Vietnam.” |
| Web/E-mail | State the type of e-survey (eg, one posted on a Web site, or one sent out through e-mail). If it is an e-mail survey, were the responses entered manually into a database, or was there an automatic method for capturing responses? | Method: Participants and Procedure | The survey was posted through various social media platforms (e.g., Facebook, Zalo, etc.).  “A cross-sectional study design was employed using a respondent driven sampling method by distributing a self-administered survey through various social media platforms (e.g., Facebook, Zalo, etc.) from April 10 to 15, 2020 in Vietnam.” |
| Context | Describe the Web site (for mailing list/newsgroup) in which the survey was posted. What is the Web site about, who is visiting it, what are visitors normally looking for? Discuss to what degree the content of the Web site could pre-select the sample or influence the results. For example, a survey about vaccination on a anti-immunization Web site will have different results from a Web survey conducted on a government Web site | Method: Participants and Procedure | The survey was posted through 2 main social media platforms: Facebook and Zalo. They are two most widespread popular social media platforms in Vietnam |
| Mandatory/voluntary | Was it a mandatory survey to be filled in by every visitor who wanted to enter the Web site, or was it a voluntary survey? | Method: Participants and Procedure | It was a completely voluntary survey.  “All Vietnamese residents who were aged 18 years and above, had signed informed consent and had voluntarily participated in the online survey during the study period were eligible for the survey. “ |
| Incentives | Were any incentives offered (eg, monetary, prizes, or non-monetary incentives such as an offer to provide the survey results)? |  | No incentives were offered for participants |
| Time/Date | In what timeframe were the data collected? | Method: Participants and Procedure | April 10 to 15, 2020 in Vietnam |
| Randomization of items or questionnaires | To prevent biases items can be randomized or alternated. |  | The survey items were not randomized |
| Adaptive questioning | Use adaptive questioning (certain items, or only conditionally displayed based on responses to other items) to reduce number and complexity of the questions. |  | This survey did not use adaptive questioning |
| Number of Items | What was the number of questionnaire items per page? The number of items is an important factor for the completion rate. |  | The questionnaire consisted of 64 questions  “The instrument is a self-administered questionnaire which consisted of three parts and could be completed in 10 minutes.” |
| Number of screens (pages) | Over how many pages was the questionnaire distributed? The number of items is an important factor for the completion rate. |  | The questionnaire spread on 4 screens |
| Completeness check | It is technically possible to do consistency or completeness checks before the questionnaire is submitted. Was this done, and if “yes”, how (usually JAVAScript)? An alternative is to check for completeness after the questionnaire has been submitted (and highlight mandatory items). If this has been done, it should be reported. All items should provide a non-response option such as “not applicable” or “rather not say”, and selection of one response option should be enforced. |  | An alternative is to check for completeness after the questionnaire has been submitted (and highlight mandatory items).  All items should provide a non-response option such as “not applicable” or “rather not say”, and selection of one response option should be enforced. |
| Review step | State whether respondents were able to review and change their answers (eg, through a Back button or a Review step which displays a summary of the responses and asks the respondents if they are correct). |  | The review of participants were not required.  A “back” button was provided if participants wished to edit previous answers. |
| Unique site visitor | If you provide view rates or participation rates, you need to define how you determined a unique visitor. There are different techniques available, based on IP addresses or cookies or both. |  | This survey did not use view rate |
| View rate (Ratio of unique survey visitors/unique site visitors) | Requires counting unique visitors to the first page of the survey, divided by the number of unique site visitors (not page views!). It is not unusual to have view rates of less than 0.1 % if the survey is voluntary. |  | We did not track how many people viewed this survey or ads |
| Participation rate (Ratio of unique visitors who agreed to participate/unique first survey page visitors) | Count the unique number of people who filled in the first survey page (or agreed to participate, for example by checking a checkbox), divided by visitors who visit the first page of the survey (or the informed consents page, if present). This can also be called “recruitment” rate. |  | We did not track this number |
| Completion rate (Ratio of users who finished the survey/users who agreed to participate) | The number of people submitting the last questionnaire page, divided by the number of people who agreed to participate (or submitted the first survey page). This is only relevant if there is a separate “informed consent” page or if the survey goes over several pages. This is a measure for attrition. Note that “completion” can involve leaving questionnaire items blank. This is not a measure for how completely questionnaires were filled in. (If you need a measure for this, use the word “completeness rate”.) | Results: Demographic characteristics | “A total of 1,412 respondents answered. From these, 1,385 (98.1%) were valid for analysis” |
| Cookies used | Indicate whether cookies were used to assign a unique user identifier to each client computer. If so, mention the page on which the cookie was set and read, and how long the cookie was valid. Were duplicate entries avoided by preventing users access to the survey twice; or were duplicate database entries having the same user ID eliminated before analysis? In the latter case, which entries were kept for analysis (eg, the first entry or the most recent)? |  | We did not use cookies to assign a unique user identifier to each client computer. |
| IP check | Indicate whether the IP address of the client computer was used to identify potential duplicate entries from the same user. If so, mention the period of time for which no two entries from the same IP address were allowed (eg, 24 hours). Were duplicate entries avoided by preventing users with the same IP address access to the survey twice; or were duplicate database entries having the same IP address within a given period of time eliminated before analysis? If the latter, which entries were kept for analysis (eg, the first entry or the most recent)? |  | The IP addresses were not collected from the participants |
| Log file analysis | Indicate whether other techniques to analyze the log file for identification of multiple entries were used. If so, please describe. |  | Other techniques to analyze the log file for identification of multiple entries were not used in this survey |
| Registration | In “closed” (non-open) surveys, users need to login first and it is easier to prevent duplicate entries from the same user. Describe how this was done. For example, was the survey never displayed a second time once the user had filled it in, or was the username stored together with the survey results and later eliminated? If the latter, which entries were kept for analysis (eg, the first entry or the most recent)? |  | This was an open survey and username login was not required for participating. |
| Handling of incomplete questionnaires | Were only completed questionnaires analyzed? Were questionnaires which terminated early (where, for example, users did not go through all questionnaire pages) also analyzed? |  | Only completed questionnaires were analyzed. |
| Questionnaires submitted with an atypical timestamp | Some investigators may measure the time people needed to fill in a questionnaire and exclude questionnaires that were submitted too soon. Specify the timeframe that was used as a cut-off point, and describe how this point was determined. |  | The duration of completing the survey was not tracked |
| Statistical correction | Indicate whether any methods such as weighting of items or propensity scores have been used to adjust for the non-representative sample; if so, please describe the methods. |  | No statistical correction methods or weighting of items were used in the analysis |

Eysenbach G. Improving the quality of Web surveys: the Checklist for Reporting Results of Internet E-Surveys (CHERRIES). J Med Internet Res. 2004 Sep 29;6(3):e34 [erratum in J Med Internet Res. 2012; 14(1): e8.]. Article available at [https://www.jmir.org/2004/3/e34](https://www.jmir.org/2004/3/e34/)/; erratum available <https://www.jmir.org/2012/1/e8/>. Copyright ©Gunther Eysenbach. Originally published in the [Journal of Medical Internet](http://www.jmir.org/) Research, 29.9.2004 and 04.01.2012.
