# Supplementary material for: Psychological Impacts of COVID-19 During the First Nationwide Lockdown in Vietnam: Web-Based, Cross-Sectional Survey Study
Source: JMIR Form Res. 2020 Dec 15;4(12):e24776. doi: 10.2196/24776 (PMC7935248; doi:10.2196/24776)
Supplement: Multimedia Appendix 2 [file formative_v4i12e24776_app2.doc]

**Multimedia Appendix 2.** Social-demographic characteristics of general population in Vietnam during the first lockdown due to COVID-19 (N = 1385).

| **Characteristics** | | **Participants** | **Characteristics** | | **Participants** |
| --- | --- | --- | --- | --- | --- |
| **Age (years), n(%)** | |  | **Having children ≤ 18 years in the family, n(%)** | |  |
|  | 18 - 39 | 1139 (82.2) |  | No | 826 (40.4) |
|  | 40 - 59 | 235 (17.0) |  | Yes | 559 (59.6) |
|  | ≥60 | 11 (0.8) | **Average income per month (million VND^a^), n(%)** | |  |
| **Gender, n(%)** | |  |  | No income | 224 (16.2) |
|  | Female | 505 (36.5) |  | < 1 | 34 (2.4) |
|  | Male | 880 (63.5) |  | 1 - <5 | 176 (12.7) |
| **Location, n(%)** | |  |  | 5 - <10 | 425 (30.8) |
|  | Rural | 374 (27.0) |  | 10 - <20 | 332 (24.0) |
|  | Urban | 1011 (73.0) |  | ≥ 20 | 193 (13.9) |
| **Marital status, n(%)** | |  | **Average time staying at home during lockdown (hours), n(%)** | |  |
|  | Single | 785 (56.7) |  | 0 - ≤ 10 | 178 (12.8) |
|  | Married | 557 (40.2) |  | 10 - ≤ 20 | 541 (39.1) |
|  | Divorced/ widowed | 43 (3.1) |  | 20 - ≤ 24 | 666 (48.1) |
| **Education level, n(%)** | |  | **Current health status, n(%)** | |  |
|  | Elementary/Secondary | 17 (1.2) |  | Very good/Good | 1254 (90.5) |
|  | High school | 194 (14.0) |  | Average | 123 (8.9) |
|  | University/ College | 918 (66.3) |  | Bad/Very bad | 8 (0.6) |
|  | Postgraduate | 256 (18.5) | **Chronic disease, n(%)** | |  |
| **Occupation, n(%)** | |  |  | No | 142 (10.3) |
|  | Employed | 449 (32.4) |  | Yes | 1243 (89.7) |
|  | Work from home | 446 (32.2) | **Live in province/ city having COVID-19 infected cases, n(%)** | |  |
|  | Student | 322 (23.2) |  | No | 966 (69.7) |
|  | Unemployed | 113 (8.2) |  | Yes | 419 (30.3) |
|  | Others | 55 (4.0) | **Current situation, n(%)** | |  |
| **Household size (member), n(%)** | |  |  | Quarantine/ Isolation | 96 (6.9) |
|  | 1 | 94 (6.8) |  | Social distancing | 1289 (93.1) |
|  | 2 | 133 (9.6) |  |  |  |
|  | 3-5 | 952 (68.7) |  |  |  |
|  | ≥6 | 206 (14.9) |  |  |  |
| ^a^ VND: Vietnam Dong | | | | | |
