# Supplementary material for: Psychological Impacts of COVID-19 During the First Nationwide Lockdown in Vietnam: Web-Based, Cross-Sectional Survey Study
Source: JMIR Form Res. 2020 Dec 15;4(12):e24776. doi: 10.2196/24776 (PMC7935248; doi:10.2196/24776)
Supplement: Multimedia Appendix 3 [file formative_v4i12e24776_app3.doc]

**Multimedia Appendix 3**. Prevalence and score of psychological and mental health impacts of COVID-19 on general population in Vietnam during the first lockdown (N= 1385).

| **Items** | **IES-R^a^** | **DASS-21^b^** | | |
| --- | --- | --- | --- | --- |
|  |  | **Depression** | **Anxiety** | **Stress** |
| Participants above cut-off point, n (%) | 497 (35.9) | 325 (23.5) | 195 (14.1) | 309 (23.3) |
| Score, Median (IQR)) | 17 (9 – 29) | 2 (0 – 8) | 0 (0 – 4) | 4 (0 -10) |
| ^a^ IES-R: Impact of Event Scale-Revised  ^b^ DASS-21: Depression, Anxiety, and Stress Scale -21 | | | | |
