# Supplementary material for: Psychological Impacts of COVID-19 During the First Nationwide Lockdown in Vietnam: Web-Based, Cross-Sectional Survey Study
Source: JMIR Form Res. 2020 Dec 15;4(12):e24776. doi: 10.2196/24776 (PMC7935248; doi:10.2196/24776)
Supplement: Multimedia Appendix 4 [file formative_v4i12e24776_app4.doc]

**Multimedia Appendix 4**. Multivariate linear regression results for Depression, Anxiety, and Stress Scale -21 Anxiety subscale with socio-demographical covariates.

| **Covariates** |  | **Coefficient (95%CI)** | ***P*** |
| --- | --- | --- | --- |
| **Age group (years) (reference: 18 – 39)** | |  |  |
|  | 40 - 59 | -0.36 (-1.21, 0.49) | .40 |
|  | ≥ 60 | -3.17 (-6.4, 0.06) | .054 |
| **Marital status (reference: Single)** | |  |  |
|  | Married | -0.36 (-1.01, 0.3) | .29 |
|  | Divorced/ widowed | 0.27 (-1.41, 1.95) | .75 |
| **Chronic disease (reference: No)** | |  |  |
|  | Yes | 0.57 (-0.38, 1.53) | .24 |
| **Current situation (reference: Social distancing)** | |  |  |
|  | Quarantine/ Isolation | 1.22 (0.11, 2.33) | .03 |
| **Current health status (reference: Very good/ Good)** | |  |  |
|  | Average | 3.64 (2.64, 4.63) | <.001 |
|  | Bad/Very bad | 7.24 (3.55, 10.94) | <.001 |
