# Supplementary material for: Psychological Impacts of COVID-19 During the First Nationwide Lockdown in Vietnam: Web-Based, Cross-Sectional Survey Study
Source: JMIR Form Res. 2020 Dec 15;4(12):e24776. doi: 10.2196/24776 (PMC7935248; doi:10.2196/24776)
Supplement: Multimedia Appendix 5 [file formative_v4i12e24776_app5.doc]

**Multimedia Appendix 5.** Multivariate linear regression results for Depression, Anxiety, and Stress Scale -21 Stress subscale with socio-demographical covariates.

| **Covariates** |  | **Coefficient (95%CI)** | ***P*** |
| --- | --- | --- | --- |
| **Occupation (reference: Employed)** | |  |  |
|  | Work from home | 0.92 (-0.03, 1.88) | .059 |
|  | Student | 1.17 (0.10, 2.24) | .03 |
|  | Unemployed | 2.34 (0.34, 3.85) | .002 |
|  | Others | 0.72 (-1.31, 2.75) | .49 |
| **Chronic disease (reference: No)** | |  |  |
|  | Yes | 1.13 (-0.16, 2.41) | .09 |
| **Current situation (reference: Social distancing)** | |  |  |
|  | Quarantine/ Isolation | 0.96 (-0.56, 2.49) | .22 |
| **Current health status (reference: Very good/ Good)** | |  |  |
|  | Average | 5.19 (3.83, 6.56) | <.001 |
|  | Bad/Very bad | 10.6 (5.56, 15.65) | <.001 |
| **Infected cases in province/ city (reference: No)** | |  |  |
|  | Yes | 0.92 (0.08, 1.76) | .03 |
