# Supplementary material for: Psychological Impacts of COVID-19 During the First Nationwide Lockdown in Vietnam: Web-Based, Cross-Sectional Survey Study
Source: JMIR Form Res. 2020 Dec 15;4(12):e24776. doi: 10.2196/24776 (PMC7935248; doi:10.2196/24776)
Supplement: Multimedia Appendix 6 [file formative_v4i12e24776_app6.doc]

**Multimedia Appendix 6**. Univariate linear regression results for Impact of Event Scale-Revised and Depression, Anxiety, and Stress Scale -21 Scale with socio-demographical covariates

| **Covariate** | | **IES-R^a^** | **DASS-21^b^** | | |
| --- | --- | --- | --- | --- | --- |
|  |  |  | **Depression** | **Anxiety** | **Stress** |
|  |  | **Coefficient**  **(95%CI)** | **Coefficient (95%CI)** | **Coefficient (95%CI)** | **Coefficient (95%CI)** |
| **Age group (years) (reference: 18 – 39)** | | | | | |
|  | 40 - 59 | 3.34 (1.26, 5.42) | -0.82 (-1.88, 0.24) | -0.49 (-1.25, 0.28) | -0.2 (-1.25, 0.85) |
|  | ≥ 60 | -4.77 (-13.56, 4.02) | -4.23 (-8.71, 0.24) | -2.66 (-5.9, 0.57) | -3.17 (-7.61, 1.27) |
|  | *P* | .004 | .06 | .13 | .36 |
| **Gender (reference: Female)** | | | | | |
|  | Male | -0.57 (-2.2, 1.05) | 0.36 (-0.46, 1.19) | 0.13 (-0.47, 0.73) | 0.18 (-0.64, 1) |
|  | *P* | .49 | .39 | .67 | .67 |
| **Location (reference: Rural)** | | | | | |
|  | Urban | 0.14 (-1.63, 1.90) | -0.19 (-1.08, 0.71) | -0.29 (-0.94, 0.35) | -0.01 (-0.9, 0.88) |
|  | *P* | .88 | .68 | .37 | .98 |
| **Marital status (reference: Single)** | | | | | |
|  | Married | 3.44 (1.84, 5.04) | -1.56 (-2.38, -0.75) | -0.62 (-1.21, -0.03) | -0.43 (-1.24, 0.38) |
|  | Divorced/ widowed | 6.68 (2.16, 11.21) | 0.43 (-1.88, 2.73) | 0.14 (-1.53, 1.81) | 0.52 (-1.77, 2.82) |
|  | *P* | <.001 | <.001 | .11 | .48 |
| **Education level (reference: Elementary/ Secondary)** | | | | | |
|  | High school | 1.44 (-5.91, 8.78) | -1.93 (-5.66, 1.81) | 0.63 (-2.07, 3.33) | -0.6 (-4.3, 3.11) |
|  | University/ College | 5.02 (-2.09, 12.12) | -2.81 (-6.42, 0.81) | 0.34 (-2.28, 2.95) | -1.38 (-4.97, 2.2) |
|  | Postgraduate | 4.10 (-3.18, 11.37) | -3.20 (-6.90, 0.51) | -0.16 (-2.83, 2.51) | -1.13 (-4.8, 2.54) |
|  | *P* | .01 | .14 | .45 | .52 |
| **Occupation (reference: Employed)** | | | | | |
|  | Work from home | -0.50 (-2.44, 1.45) | 1.33 (0.35, 2.31) | 0.12 (-0.59, 0.84) | 0.93 (-0.05, 1.91) |
|  | Student | -2.98 (-5.10, -0.86) | 2.11 (1.04, 3.18) | 0.64 (-0.14, 1.42) | 1.04 (-0.03, 2.11) |
|  | Unemployed | 1.13 (-1.93, 4.18) | 3.60 (2.06, 5.15) | 0.83 (-0.3, 1.95) | 2.3 (0.76, 3.83) |
|  | Others | 0.11 (-4.04, 4.26) | 1.24 (-0.86, 3.33) | -0.13 (-1.65, 1.4) | 0.89 (-1.2, 2.98) |
|  | *P* | .03 | <.001 | .36 | .004 |
| **Household size (member) (reference: 1)** | | | | | |
|  | 2 | 4.73 (0.81, 8.65) | 4.73 (0.81, 8.65) | -0.13 (-1.57, 1.31) | 0.22 (-1.75, 2.2) |
|  | 3-5 | 2.64 (-0.50, 5.79) | 2.64 (-0.50, 5.79) | 0.01 (-1.14, 1.17) | 0.41 (-1.17, 2) |
|  | ≥ 6 | 3.02 (-0.60, 6.64) | 3.02 (-0.60, 6.64) | -0.39 (-1.72, 0.94) | -0.11 (-1.93, 1.72) |
|  | *P* | .13 | .65 | .81 | .80 |
| **Having children ≤ 18 years old in the family (reference: No)** | | | | | |
|  | Yes | 1.44 (-0.15, 3.03) | -0.48 (-1.29, 0.33) | -0.2 (-0.78, 0.39) | 0.11 (-0.69, 0.91) |
|  | *P* | .08 | .24 | .51 | .79 |
| **Chronic disease (reference: No)** | | | | | |
|  | Yes | 3.22 (0.65, 5.80) | 1.51 (0.21, 2.82) | 0.89 (-0.06, 1.83) | 1.74 (0.44, 3.03) |
|  | *P* | .01 | .02 | .07 | .009 |
| **Current situation (reference: Social distancing)** | | | | | |
|  | Quarantine/ Isolation | 1.24 (-1.84, 4.32) | 1.42 (-0.15, 2.98) | 1.25 (0.12, 2.38) | 1.26 (-0.29, 2.81) |
|  | *P* | .43 | .08 | .03 | .11 |
| **Average income per month (million VND^c^) (reference: No income)** | | | | | |
|  | <1 | 2.91 (-2.44, 8.25) | 0.54 (-2.18, 3.26) | 1.2 (-0.77, 3.16) | 0.76 (-1.94, 3.46) |
|  | 1-5 | 1.29 (-1.63, 4.22) | -1.22 (-2.71, 0.26) | -0.83 (-1.9, 0.25) | -0.97 (-2.45, 0.5) |
|  | 5-10 | 3.89 (1.49, 6.29) | -1.20 (-2.42, 0.02) | -0.26 (-1.14, 0.62) | -0.68 (-1.89, 0.53) |
|  | 10-20 | 1.52 (-0.99, 4.03) | -1.80 (-3.08, -0.53) | -0.75 (-1.67, 0.17) | -0.94 (-2.21, 0.32) |
|  | >20 | 2.82 (-0.03, 5.68) | -1.26 (-2.71, 0.19) | -0.48 (-1.53, 0.56) | -0.18 (-1.62, 1.26) |
|  | *P* | .03 | .09 | .23 | .50 |
| **Average time stays at home during social distancing(reference: 0-10 hour(s))** | | | | | |
|  | 10-20 hours | -0.50 (-3.01, 2.01) | 0.03 (-1.24, 1.31) | -0.63 (-1.55, 0.29) | -0.16 (-1.43, 1.1) |
|  | 20-24 hours | -2.52 (-4.97, -0.07) | 1.25 (0.01, 2.50) | -0.64 (-1.55, 0.26) | 0.06 (-1.18, 1.3) |
|  | *P* | .03 | .01 | .34 | .87 |
| **Current health status (reference: Very good/ Good)** | | | | | |
|  | Average | 9.06 (6.35, 11.76) | 6.20 (4.84, 7.55) | 3.7 (2.72, 4.69) | 5.46 (4.12, 6.81) |
|  | Bad/Very bad | 7.67 (-2.50, 17.84) | 9.34 (4.25, 14.42) | 7.26 (3.57, 10.96) | 10.34 (5.28, 15.4) |
|  | *P* | <.001 | <.001 | <.001 | <.001 |
| **Infected cases in province/ city (reference: No)** | | | | | |
|  | Yes | 1.25 (-0.45, 2.95) | 1.51 (0.65, 2.38) | 0.38 (-0.24, 1.01) | 0.94 (0.08, 1.8) |
|  | *P* | .15 | <.001 | .23 | .03 |
| ^a^ IES-R: Impact of Event Scale-Revised  ^b^ DASS-21: Depression, Anxiety, and Stress Scale -21  ^c^ VND: Vietnam Dong | | | | | |
