# Supplementary material for: Psychological Impacts of COVID-19 During the First Nationwide Lockdown in Vietnam: Web-Based, Cross-Sectional Survey Study
Source: JMIR Form Res. 2020 Dec 15;4(12):e24776. doi: 10.2196/24776 (PMC7935248; doi:10.2196/24776)
Supplement: Multimedia Appendix 7 [file formative_v4i12e24776_app7.doc]

**Multimedia Appendix 7.** Univariate linear regression results for Impact of Event Scale-Revised and Depression, Anxiety, and Stress Scale -21 with concern-related covariates.

| **Covariates** | | **IES-R^a^** | **DASS-21^b^** | | |
| --- | --- | --- | --- | --- | --- |
|  |  |  | **Depression** | **Anxiety** | **Stress** |
|  |  | **Coefficient (95%CI)** | **Coefficient (95%CI)** | **Coefficient (95%CI)** | **Coefficient (95%CI)** |
| **Level of confidence in own doctor’s ability to diagnose, treat (reference: Do not know)** | |  |  |  |  |
|  | Very confident | -7.84 (-14.58, -1.11) | -2.07 (-5.49, 1.35) | -0.79 (-3.26, 1.67) | -2.23 (-5.62, 1.15) |
|  | Somewhat confident | -5.57 (-12.33, 1.20) | -0.44 (-3.87, 3.00) | 0.35 (-2.12, 2.83) | -0.59 (-3.99, 2.81) |
|  | Not very confident | -3.82 (-12.5, 4.87) | -0.32 (-4.73, 4.09) | 1.82 (-1.36, 5.00) | 0.24 (-4.12, 4.61) |
|  | Not at all confident | -0.63 (-16.59, 15.33) | 1.16 (-6.94, 9.26) | -1.87 (-7.71, 3.97) | -3.18 (-11.2, 4.83) |
|  | *P* | .009 | .002 | <.001 | .001 |
| **Likelihood of contracting COVID−19 during the current outbreak (reference: Do not know)** | |  |  |  |  |
|  | Very likely | 3.85 (-0.32, 8.01) | -0.18 (-2.32, 1.95) | 0.90 (-0.64, 2.43) | 1.26 (-0.85, 3.36) |
|  | Somewhat likely | 2.61 (-0.81, 6.03) | 1.15 (-0.60, 2.90) | 1.06 (-0.20, 2.32) | 1.61 (-0.12, 3.34) |
|  | Not very likely | -1.52 (-4.74, 1.70) | -0.29 (-1.94, 1.36) | -0.39 (-1.57, 0.80) | 0.26 (-1.36, 1.89) |
|  | Not at all likely | -3.69 (-7.26, -0.12) | -1.10 (-2.94, 0.73) | -0.99 (-2.31, 0.32) | -1.26 (-3.07, 0.55) |
|  | *P* | <.001 | .01 | <.001 | <.001 |
| **Likelihood of surviving if infected with COVID-19 (reference: Do not know)** | |  |  |  |  |
|  | Very likely | -3.87 (-6.41, -1.33) | 0.08 (-1.22, 1.37) | -0.31 (-1.25, 0.63) | -0.70 (-1.98, 0.59) |
|  | Somewhat likely | 1.91 (-0.79, 4.60) | 2.33 (0.96, 3.71) | 1.47 (0.47, 2.47) | 1.79 (0.43, 3.16) |
|  | Not very likely | 6.28 (1.26, 11.31) | 4.58 (2.01, 7.15) | 1.58 (-0.28, 3.44) | 2.99 (0.45, 5.54) |
|  | Not at all likely | -0.61 (-17.27, 16.05) | 9.41 (0.9, 17.92) | 5.24 (-0.92, 11.39) | 2.73 (-5.71, 11.17) |
|  | *P* | <.001 | <.001 | <.001 | <.001 |
| **Concerns about a child younger than 18 years getting COVID-19 infection (reference: Have no children <18 years old)** | |  |  |  |  |
|  | Very worried | 10.69 (8.18, 13.19) | 0.53 (-0.80, 1.87) | 1.22 (0.26, 2.17) | 2.28 (0.96, 3.59) |
|  | Somewhat worried | 4.73 (2.27, 7.19) | -0.84 (-2.15, 0.47) | 0.14 (-0.8, 1.08) | 0.74 (-0.55, 2.03) |
|  | Not very worried | 1.09 (-1.43, 3.62) | -0.74 (-2.08, 0.61) | -0.38 (-1.34, 0.59) | 0.13 (-1.20, 1.45) |
|  | Not at all worried | -4.1 (-7.51, -0.68) | -1.41 (-3.23, 0.40) | -1.17 (-2.47, 0.14) | -0.83 (-2.62, 0.97) |
|  | *P* | <.001 | .04 | <.001 | <.001 |
| **Concerns about other family members getting COVID−19 infection (reference: Have no family member)** | |  |  |  |  |
|  | Very worried | 6.15 (-10.09, 22.39) | 0.93 (-7.63, 9.49) | 0.67 (-5.49, 6.82) | -3.92 (-12.36, 4.51) |
|  | Somewhat worried | 0.75 (-15.48, 16.97) | -0.04 (-8.59, 8.51) | -0.38 (-6.53, 5.77) | -5.22 (-13.64, 3.21) |
|  | Not very worried | -3.15 (-19.39, 13.08) | 0.11 (-8.45, 8.66) | -0.64 (-6.8, 5.51) | -5.78 (-14.22, 2.65) |
|  | Not at all worried | -8.32 (-24.83, 8.19) | -1.81 (-10.52, 6.89) | -1.53 (-7.79, 4.73) | -7.45 (-16.03, 1.13) |
|  | *P* | <.001 | .06 | .002 | <.001 |
| ^a^ IES-R: Impact of Event Scale-Revised  ^b^ DASS-21: Depression, Anxiety, and Stress Scale -21 | | | | | |
