# Supplementary material for: Psychological Impacts of COVID-19 During the First Nationwide Lockdown in Vietnam: Web-Based, Cross-Sectional Survey Study
Source: JMIR Form Res. 2020 Dec 15;4(12):e24776. doi: 10.2196/24776 (PMC7935248; doi:10.2196/24776)
Supplement: Multimedia Appendix 8 [file formative_v4i12e24776_app8.doc]

**Multimedia Appendix 8.** Multivariate linear regression results for Impact of Event Scale-Revised and Depression, Anxiety, and Stress Scale -21 with concern-related covariates.

| **Covariates** | | **IES-R** | | **DASS-21** | | | | | |
| --- | --- | --- | --- | --- | --- | --- | --- | --- | --- |
|  |  |  |  | **Depression** | | **Anxiety** | | **Stress** | |
|  |  | **Coefficient (95%CI)** | ***P*** | **Coefficient (95%CI)** | ***P*** | **Coefficient (95%CI)** | ***P*** | **Coefficient (95%CI)** | ***P*** |
| **Level of confidence in own doctor’s ability to diagnose, treat (reference: Do not know)** | |  |  |  |  |  |  |  |  |
|  | Very confident | -4.82 (-11.31, 1.67) | .15 | -1.42 (-4.88, 2.04) | .42 | -0.20 (-2.69, 2.29) | .88 | -1.47 (-4.89, 1.94) | .40 |
|  | Somewhat confident | -2.69 (-9.2, 3.81) | .42 | -0.11 (-3.57, 3.36) | .95 | 0.78 (-1.71, 3.28) | .54 | -0.12 (-3.54, 3.30) | .95 |
|  | Not very confident | -2.51 (-10.82, 5.80) | .55 | -0.55 (-4.98, 3.88) | .81 | 1.86 (-1.33, 5.04) | .25 | 0.15 (-4.22, 4.52) | .95 |
|  | Not at all confident | 1.48 (-13.68, 16.64) | .85 | 1.55 (-6.53, 9.62) | .71 | -1.16 (-6.96, 4.65) | .70 | -2.69 (-10.66, 5.29) | .51 |
| **Likelihood of contracting COVID−19 during the current outbreak (reference: Do not know)** | |  |  |  |  |  |  |  |  |
|  | Very likely | 1.14 (-3.07, 5.35) | .59 | -0.84 (-3.08, 1.40) | .46 | 0.32 (-1.29, 1.93) | .69 | 0.51 (-1.71, 2.72) | .65 |
|  | Somewhat likely | 2.65 (-0.87, 6.18) | .14 | 0.77 (-1.11, 2.65) | .42 | 0.75 (-0.60, 2.10) | .28 | 1.37 (-0.48, 3.22) | .15 |
|  | Not very likely | 0.14 (-3.19, 3.47) | .93 | -0.36 (-2.13, 1.42) | .69 | -0.39 (-1.66, 0.89) | .55 | 0.48 (-1.27, 2.23) | .59 |
|  | Not at all likely | -0.35 (-4.01, 3.31) | .85 | -0.62 (-2.57, 1.33) | .54 | -0.60 (-2.00, 0.80) | .40 | -0.49 (-2.42, 1.43) | .61 |
| **Likelihood of surviving if infected with COVID-19 (reference: Do not know)** | |  |  |  |  |  |  |  |  |
|  | Very likely | -1.83 (-4.48, 0.82) | .17 | 0.28 (-1.13, 1.69) | .70 | 0.00 (-1.02, 1.01) | .99 | -0.39 (-1.78, 1.01) | .59 |
|  | Somewhat likely | 1.54 (-1.24, 4.31) | .28 | 2.07 (0.59, 3.55)** | .01 | 1.21 (0.15, 2.27)* | .03 | 1.31 (-0.15, 2.77) | .08 |
|  | Not very likely | 3.72 (-1.21, 8.64) | .24 | 4.10 (1.47, 6.72)** | .002 | 1.00 (-0.89, 2.89) | .30 | 2.22 (-0.37, 4.81) | .09 |
|  | Not at all likely | 1.49 (-14.51, 17.49) | .86 | 8.67 (0.14, 17.19)* | .05 | 5.2 (-0.93, 11.33) | .10 | 2.74 (-5.68, 11.15) | .52 |
| **Concerns about a child younger than 18 years getting COVID-19 infection (reference: Have no children <18 years old)** | |  |  |  |  |  |  |  |  |
|  | Very worried | 7.81 (4.98, 10.64) | <.001 | 0.23 (-1.28, 1.74) | .76 | 0.89 (-0.20, 1.97) | .11 | 1.75 (0.26, 3.24) | .02 |
|  | Somewhat worried | 3.19 (0.61, 5.78) | .02 | -1.15 (-2.53, 0.23) | .10 | -0.02 (-1.01, 0.97) | .97 | 0.31 (-1.05, 1.67) | .65 |
|  | Not very worried | 1.71 (-0.84, 4.25) | .19 | -0.70 (-2.06, 0.65) | .31 | -0.18 (-1.16, 0.79) | .71 | 0.31 (-1.02, 1.65) | .64 |
|  | Not at all worried | -1.81 (-5.38, 1.76) | .32 | -0.68 (-2.58, 1.22) | .48 | -0.80 (-2.17, 0.57) | .25 | 0.03 (-1.85, 1.91) | .98 |
| **Concerns about other family members getting COVID−19 infection (reference: Have no family member)** | |  |  |  |  |  |  |  |  |
|  | Very worried | 2.64 (-13.29, 18.58) | .67 | 1.33 (-7.17, 9.82) | .76 | 0.84 (-5.26, 6.94) | .79 | -4.18 (-12.55, 4.20) | .33 |
|  | Somewhat worried | 0.25 (-15.63, 16.13) | .89 | 1.02 (-7.44, 9.48) | .81 | 0.36 (-5.72, 6.44) | .91 | -4.68 (-13.03, 3.66) | .27 |
|  | Not very worried | -1.14 (-17.04, 14.77) | .98 | 1.31 (-7.17, 9.78) | .76 | 0.60 (-5.49, 6.69) | .85 | -4.71 (-13.07, 3.66) | .27 |
|  | Not at all worried | -3.57 (-19.84, 12.70) | .75 | -0.09 (-8.76, 8.57) | .98 | 0.34 (-5.89, 6.57) | .91 | -5.50 (-14.05, 3.05) | .21 |
| ^a^ IES-R: Impact of Event Scale-Revised  ^b^ DASS-21: Depression, Anxiety, and Stress Scale -21 | | | | | | | | | |
